# Violet Light Effects on the Circadian Rest–Activity Rhythm and the Visual System

**DOI:** 10.3390/clockssleep6030029

**Published:** 2024-08-14

**Authors:** Teresa Domínguez-Valdés, Cristina Bonnin-Arias, Cristina Alvarez-Peregrina, Beatriz G. Galvez, Miguel Angel Sanchez-Tena, Francisco Germain, Pedro de la Villa, Celia Sánchez-Ramos

**Affiliations:** 1 Faculty of Optics and Optometry, Universidad Complutense de Madrid, 28037 Madrid, Spain; matedomi@ucm.es (T.D.-V.); cbonnina@ucm.es (C.B.-A.); cristina_alvarez@ucm.es (C.A.-P.); celiasr@opt.ucm.es (C.S.-R.); 2 Vision and Ophthalmology Research Group, Universidad Complutense de Madrid, 28037 Madrid, Spain; 3 Department of Biochemistry and Molecular Biology, Faculty of Pharmacy, Universidad Complutense de Madrid, 28040 Madrid, Spain; bggalvez@ucm.es; 4 ISEC LISBOA-Instituto Superior de Educação e Ciências, 1750-179 Lisbon, Portugal; 5 Department of System Biology, University of Alcalá, 28805 Alcalá de Henares, Spain; francisco.germain@uah.es (F.G.); pedro.villa@uah.es (P.d.l.V.); 6 Visual Neurophysiology Group, Instituto Ramon y Cajal de Investigación Sanitaria (IRYCIS), 28034 Madrid, Spain

**Keywords:** violet light, circadian cycle, visual function, Snoezelen rooms

## Abstract

Background: Rooms illuminated by “black light” (<400 nm wavelength) has become popular, but there is not enough scientific evidence to support its implementation. This study aims to assess the effects of violet light (392 nm) on the circadian rest–activity rhythm and the visual system through animal experimentation. Materials and results: Five groups of four mice were exposed to different white light, violet light, and dark periods, and their circadian rhythm was analyzed by measuring the circadian period using rest–activity cycles. Electroretinographic recordings and structural analysis of the retina were also performed on experimental animals. Results: Our study demonstrates that mice present normal circadian activity during exposure to violet light, taking rest not only under white light but under violet lighting periods. However, mice suffered a decrease in electrical retinal response after exposure to violet light as measured by electroretinography. Nevertheless, no structural changes were observed in the retinas of the animals under different lighting conditions. Conclusions: Violet light elicits circadian rest–activity rhythm in mice but alters their visual function, although no structural changes are observed after short periods of violet light exposure.

## 1. Introduction

The use of sensory stimulation rooms is widely established for the improvement of people with sensory problems. In this sense, Snoezelen multi-sensory environments can be used by all ages and abilities, highlighting the benefits of their implementation in the first years of life, in challenging behaviors, occupational therapy, learning disabilities, mental health, and patients with autism, brain injuries, or in the last years of life [1,2].

Among the elements included in this type of room, light, specifically Blue/Violet light, plays an essential role. Blue/Violet light is defined as a light source with wavelengths between 350 and 392 nm within the spectrum that includes, in some cases, UVA radiation from 315 to 380 nm. Thus, in these rooms, light stimuli are used to experience “the magic of Blue/Violet Light” [2]. This type of light is further used for the inspection of sanitary rooms or operating rooms, or for other uses of interest. Regarding the application of ophthalmology, it is used in the autofluorescence of the eye fundus, allowing for the detection of retinal pathologies [3,4].

Numerous scientific studies conducted in the past have demonstrated that prolonged exposure to high levels of Blue/Violet light can elicit adverse effects on the human eye, which may be associated with the development of age-related macular degeneration (AMD) [5,6]. Recent research has substantiated previous findings, revealing that exposure to Blue/Violet light can increase the risk of AMD development, as evidenced in both animal [7] and human models [8].

As far as education is concerned, the Snoezelen rooms illuminated with Blue/Violet light (known as “Black light”) have been implemented to help the development of children in special education centers, although most of the teachers declare that they are unaware of the scientific evidence on which their implementation is based [9]. In recent years, Blue/Violet light rooms have expanded in kindergartens and schools, both in special education and regular education. The few articles published about the use of these rooms in schools conclude that more research is needed to support their installation in educational centers [9].

The influence of light of different wavelengths on our biological clock has been extensively studied. The vertebrate retina comprises visual photoreceptors called rods and cones, which are responsible for color and dark vision. In addition, the inner retina also contains intrinsically photosensitive retinal ganglion cells (ipRGCs) that detect blue light and express melanopsin, a photopigment that plays a role in synchronizing the retinal clock [10]. The fact that melanopsin can play an important role in circadian rhythms opens a new research area to design experiments comparing the effect of different wavelengths.

Added to the few contributions in the scientific literature found for its implementation in schools is the lack of research studies on the risks of exposure to Blue/Violet light. This work aims to know the possible damage that this light could induce on the circadian rest–activity cycle and the visual system through animal (mouse) experimentation. 

Previous studies addressed the light perception in mice (see review [11]). In the mouse, responses to light are mediated by retinal photoreceptors (rods and cones), as well as the melanopsin-expressing photosensitive retinal ganglion cells (psRGCs). Furthermore, one of the two types of cone cells in the mouse retina is sensitive to UV and can regulate circadian rhythms [12]. In contrast to other mammals, the lens of the mouse eye does not filter UV light, allowing for the detection of light with wavelengths in the UVA range [13]. 

Different protocols of white and violet light (392 nm peak emission wavelength) have been used (see Section 4) in a series of experiments carried out to test circadian rest–activity rhythms. Moreover, the effect of such violet light on retinal function has been tested by electroretinographic recording. Finally, the structure of the retina of the animals maintained under violet light cycles has been addressed by immunohistochemistry. 

## 2. Results

### 2.1. Violet Light Directly Impacts the Circadian Rest–Activity Rhythm of Mice

**Figure 1:**
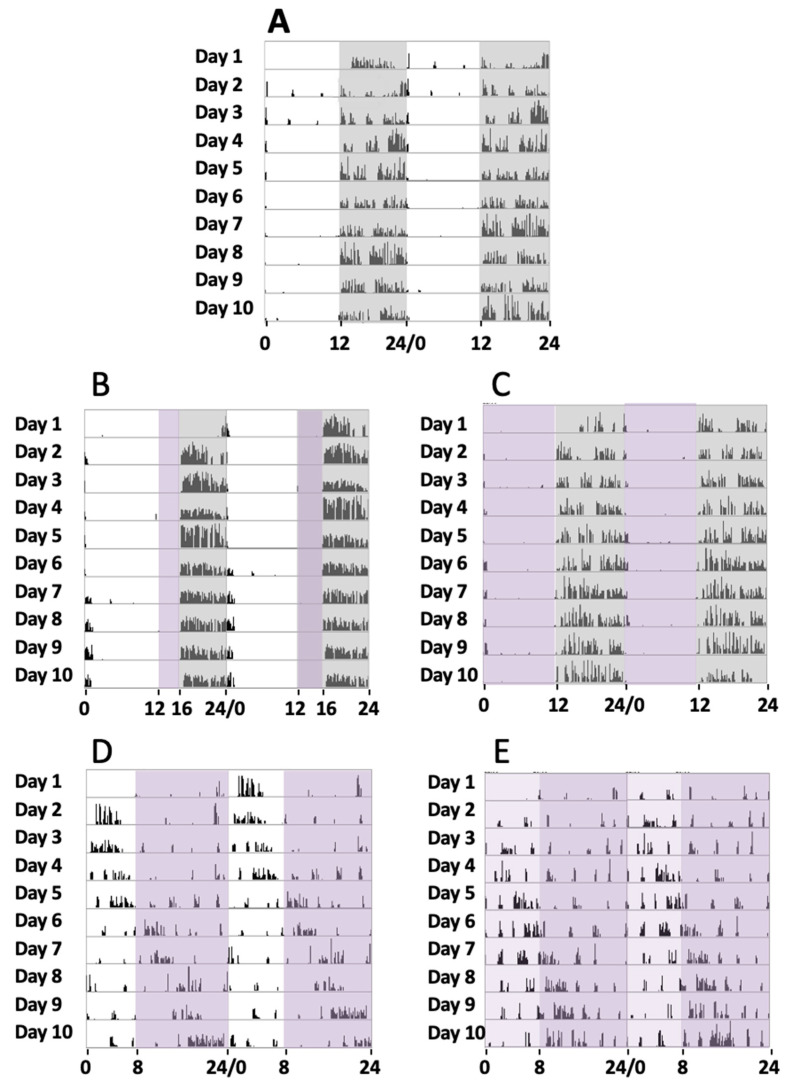
Locomotor circadian activity. Representative actograms of the locomotor activity of 5 mice groups over 10 days of continued recording in different experimental conditions (days 11 to 20 of the 30 days recorded). The number of wheel rounds was measured every 10 min and two days of consecutive activity are shown in each row. (**A**) Here, 12 h white light/12 h dark (grey shadow). (**B**) Here, 12 h white light, 4 h violet light (purple shadow), and 8 h dark. (**C**) Here, 12 h violet light and 12 h dark. (**D**) Here, 8 h white light and 16 h violet light. (**E**) Here, 8 h white plus violet lights (pale purple shadow) and 16 h violet light.

Initially, a series of four animals were maintained under a normal circadian rhythm with white light and dark exposure periods of 12 h/12 h, respectively, for 30 days (group 1). During the experiments, a record of their activity from the sleep–wake periods was addressed by the actogram cages. Locomotor activity measurement under such conditions showed that the animals were active during the dark period and took rest during the light exposure (Figure 1A), with a circadian period of 24.23 ± 0.34 h (mean, SD). 

In a second series of experiments, a group of four mice were exposed to white light, violet radiation, and darkness in cycles of 12 h/4 h/8 h, respectively, for 30 days (group 2). In these cases, their sleep–wake cycles could be recorded, and it could be observed that during the violet light period, mice showed a clear tendency to inactivity and even to sleep since no locomotor activity was observed. In turn, in the dark period, they tended to run on the activity wheel (Figure 1B), with a circadian period of 23.91 ± 0.46 h (mean, SD). These experiments suggested that the animals remained inactive in the violet light period. To completely address the effect of violet light, we conducted a further series of experiments.

In a third series of experiments, a group of four mice was exposed to violet radiation and darkness in cycles of 12 h/12 h, respectively, for 30 days (group 3). The mice were exposed to violet radiation and darkness in cycles of 12 h/12 h, respectively. We could observe that the animals did not maintain any activity during the 12 h violet phase (Figure 1C), just like under white light. The animal became active just after the violet light was off and the dark period started. A circadian period of 24.16 ± 0.31 h was measured (mean, SD). Comparative analyses of the circadian periods between groups 1, 2, and 3 did not show significant statistical differences (G1 vs G2, G1 vs G3, and G2 vs G3, one-way ANOVA, Sidak’s multiple comparisons test, *p* > 0.9999, 0.6179, and 0.6212, respectively).

In a fourth series of experiments, another group of four mice were exposed to white light, and violet radiation in cycles of 8 h/16 h, respectively (group 4). Measurements of their circadian activity were taken over 30 days. In these experiments, substantial differences were observed when compared with experiments that included a dark phase. We could observe that the animals showed abnormal circadian active phases. They experience some hours of active behaviors followed by hours of inactivity, with a circadian period of 25.57 ± 0.87 h (mean, SD) (Figure 1D). Comparative analyses between group 4 and the groups that include a dark phase (groups 1, 2, and 3) showed statistically significant differences (one-way ANOVA, Sidak’s multiple comparisons test, *p* < 0.0001).

Finally, in a fifth series of experiments, another group of four mice was exposed to a phase of white light plus violet radiation followed by a phase of violet light in cycles of 8 h/16 h, respectively, for 30 days (group 5). As expected, these experiments demonstrated similar results to the fourth group, since the period of activity and rest were not concentrated into a special light-illuminating phase, and a circadian period of 25.43 ± 0.60 h (mean, SD) was measured. Comparative analyses between group 5 and the groups that include a dark phase (groups 1, 2, and 3) showed statistically significant differences (one-way ANOVA, Sidak’s multiple comparisons test, *p* < 0.0001), but no differences were observed with group 4 (*p* = 0.99). 

Figure 1 shows the actograms of the five animal groups over 10 days. Although circadian activity was tested for 30 days, just the activity from day 11 until day 20 is shown, once the activity record was stable enough after starting lighting conditions for each group. 

### 2.2. Violet Radiation Induces Functional Changes in the Visual System

**Figure 2:**
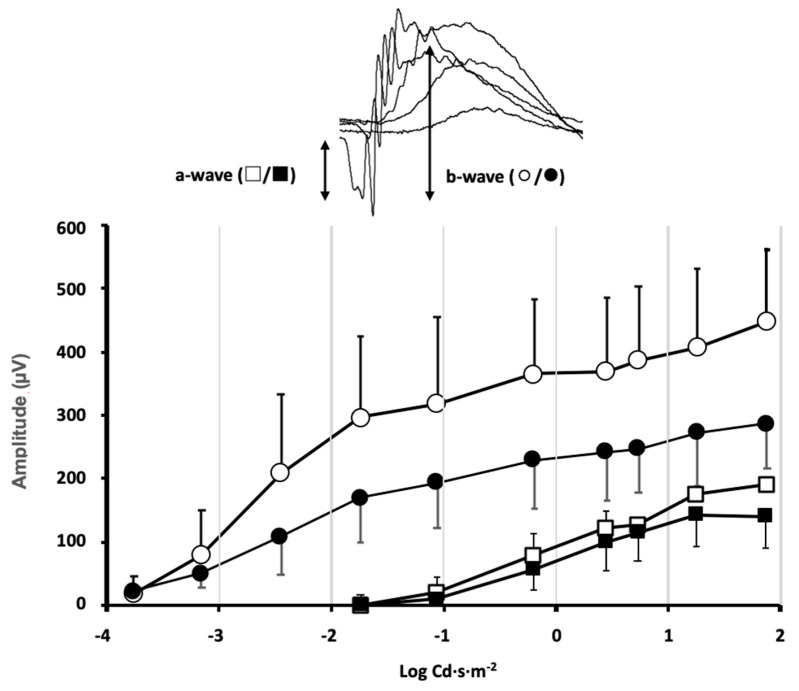
Effect of violet light on retinal function evaluated by flash ERG.

Animals from different experimental groups were subjected to electroretinogram tests (ERG) to determine the state of retinal function and the possible impact that exposure to violet light could have. Most animal groups did not show significant differences between the ERG recorded in control conditions, before locomotor activity measurement, and at the end of the 30-day period of different light exposures. However, in the group maintained under constant violet light exposure (group 5), a significant decrease in the ERG wave amplitudes could be observed (Figure 2). The mice maintained under constant violet light exposure (a phase of white light plus violet radiation followed by a phase of violet light in cycles of 8 h/16 h, respectively) experienced a decrease in the ERG wave amplitudes. The amplitude of the a-wave was slightly reduced in response to high-intensity stimuli although no significant differences were observed when comparing ERG a-wave amplitude before and after violet light exposure (*p* = 0.34, two-way ANOVA). However, a significant decrease in the b-wave amplitude could be observed for ERG b-wave amplitude before and after violet light exposure (*p* = 0.012, two-way ANOVA). According to the origin of the ERG waves, this amplitude decrease could be related not only to the effect of violet light on photoreceptor sensitivity but also to its effect on the synaptic signal amplification between photoreceptors and rod bipolar cells. 

Stimulus–response curves of the electroretinographic wave amplitudes were measured in the mice of group 5, under control conditions of 12 h light/12 h dark cycles (◯/□ open symbols) and the same mice after exposure to 8 h white plus violet light/16 h violet light cycles (●/■ closed symbols) for 30 days. *Inset*: The a-wave deflection induced by photoreceptor hyperpolarization is measured from the baseline to the peak negative deflection. The b-wave induced by second-order neurons is measured from the peak negative deflection to the peak positive deflection. A significant decrease in the b-wave amplitude was induced by the violet light (data shown correspond to mean ± SD, *n* = 4, *p* = 0.012, two-way ANOVA). 

### 2.3. Violet Radiation Does Not Produce Changes in the Structure of the Retinas of Exposed Animals

**Figure 3:**
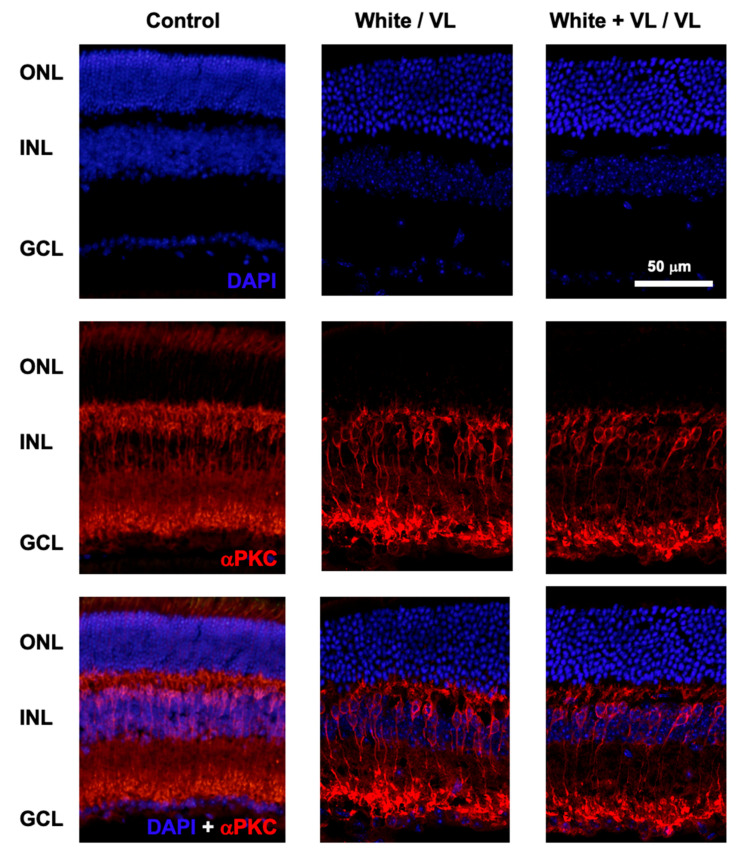
Retinal structure after violet light exposure. Representative images of retinal sections from mice maintained under different light conditions. Control: retinal sections of an animal maintained under 12 h white light/12 h dark phase periods. White/VL: retinal sections of an animal maintained under 8 h white light/16 h violet light phase periods. White + VL/VL: retinal sections of an animal maintained under 8 h white plus violet lights/16 h violet light phase periods. Retinal cells were labeled with DAPI: nuclear labeling (upper row), and αPKC immunostaining: Rod bipolar cells (middle row). Double labeled with DAPI and αPKC is shown (lower row). Retinal nuclear layers are indicated on the left: ONL: Outer Nuclear Layer. INL: Inner Nuclear Layer. GCL Ganglion Cell Layer.

At the end of the experimental test of locomotor activity, animals of the different groups were sacrificed, and the retinas were processed for histological studies. The putative effect of different light exposures on the number of retinal cells was studied by DAPI labeling of cell nuclei, and αPKC staining of rod bipolar cells (Figure 3). 

No structural changes were observed in the retinas of the animals of different experimental groups under any of the light exposure conditions observed in comparison with their controls. The confocal images of retinal sections did not show any structural modifications in the retinal cell number (DAPI staining) from those groups exposed to the longest periods of violet light exposure (8 h white light/16 h violet light cycles and 8 h white light plus violet light/16 h violet light cycles). The number of cell somas per column was measured in the control animals of group 1 (12.35 ± 0.97, mean ± SD, n = 4), group 4 (12.17 ± 1.43, mean ± SD, n = 4), and group 5 (12.14 ± 1.37, mean ± SD, n = 4). No significant differences were observed between groups (group 1 vs. group 4, *p* = 0.56, and group 1 vs. group 5, *p* = 0.35, *t*-test). 

Similarly, no apparent significant changes in the number and structure of rod bipolar cells could be observed among groups. 

## 3. Discussion

The expansion of rooms in schools illuminated with lights under a 400 nm wavelength and the little scientific research about how it can impact the visual system of children make it important to carry out studies that let us know if these light sources alter the circadian rhythms or cause damage in the retina.

To our knowledge, this is the first study that mimics various scenarios of rooms illuminated with light sources under visible spectrum (<400 nm) to evaluate the effect on the mouse retina and circadian rest–activity rhythm. The results show an alteration in the circadian rhythm and the visual function, not finding differences in the retina structure.

Our study, comparing the effect of white lights, with an emission curve with two maximum peaks at 449 and 543 nm, to the violet radiation, with an emission curve with a peak at 392 nm on rest–activity circadian rhythm, shows similar results, confirming the influence of the exposure to violet lights on circadian rhythm in mice. Our results are in accordance with previous results obtained in mice, showing that their cones are sensitive to UV light, and the absence of UV filtering by the mouse lens determines the cone-mediated regulation of circadian rhythms [12,13,14]. Moreover, the illuminance of the violet light we use in our experiments (16 lux) is equivalent to the light intensity (12.9 Log photons/cm^2^/s) used for circadian entrainment in previous works [14]. 

Human beings are not able to perceive ultra-violet light because the peak sensitivity of the short wavelength cones in a human is about 420 nm and ultra-violet light is very poorly transmitted to the retina: less than 1% UVA transmission and no UVB transmission [15,16]. It remains to be determined if violet light with a peak sensitivity of 392 nm is also able to entrain humans into circadian rhythms like in mice.

Not only wavelength composition is important in the circadian rhythm, but it depends also on the timing and intensity of the exposure. So, light exposure in the evening, at night, and in the morning affected the circadian phase of melatonin levels [17]. 

Another important effect that short wavelengths can have on the retina is related to the visual function. There are several ways to measure visual function, with electroretinography being one of the most reliable, although it cannot be translated easily to our daily clinic [18]. Our study shows how visual function is altered when mice are exposed to violet light, losing the retinal response after exposure. In addition, the study indicates that longer exposure to short wavelengths produces greater changes in the visual function, with an important decrease in retinal electrical signals after just four weeks of exposure.

Some studies have shown how violet light can influence visual function with different results. For instance, Lou et al. showed how long-term violet light did not affect in vivo retinal function in young rhesus monkeys [19]. On the other side, Narimatsu et al. reported the worst electroretinogram response after violet light exposure in mice [20]. These differences may be attributed to the different animal species used. Moreover, research published about the ocular damaging effects of violet light exposure focused on the electrophysiology of the retina gave the same result, a decrease in the electroretinogram after violet light exposure [21,22,23,24]. 

Regarding other wavelengths different from violet light, Zhang et al. showed how exposure to short-wavelength artificial light in the early stage of vision-dependent development in mice delayed the development of the visual pathway [25]. This result is important for our research as far as our main concern, which is the damage that exposure to violet light could have on children. 

Finally, this research evaluated changes in the retinal structure and found that there are no changes after four weeks of violet light exposure. These findings can be due to this short period of one month since the changes in the visual function probably come from molecular changes in the retinal cells. As found in the scientific literature, three mechanisms are implicated in the damage to the retina produced by light. The first was the photomechanical damage [26], followed by the photothermal [27] and photochemical one [28,29,30,31], and it is known that short wavelengths affect the retina. 

In the narrative review about ocular hazards and the prevention of violet light exposure, Cougnard-Gregoire et al. state how this kind of light with high energy induces and accelerates photochemical reactions and cellular damage, contributing to the loss of photoreceptors, lipid peroxidation, and cell apoptosis [32].

This study presents some limitations that require discussion. The experimental duration is relatively short, precluding the observation of any structural changes despite the findings concerning increased circadian activity and the loss of retinal response in mice subjected to violet light. It would be interesting in future studies to compare the phase, amplitude, and period parameters of two scenarios for statistically significant differences and circadian patterns of activity. On the other side, there are differences between human and mouse retinas that make this study unable to be extrapolated to humans. Further research needs to be conducted to confirm the effects on humans, especially in children, but based on these results, some prevention measures should be taken. 

## 4. Materials and Methods

### 4.1. Animals and Ethics Procedures

All experimental scientific procedures were carried out following the European regulations (Directive 86/609/CEE) and national laws (Royal Decree 53/2013) to protect experimental animals, as well as Law 32/2007 for the care of animals in all phases of research. Also, the investigation has conformed to the Statement for the Use of Animals in Vision Research (ARVO 2013). Experimentation was performed with the Authorization of the Animal Experimentation Committee and the Community of Madrid for the study of animal experimentation (ES280790001941). Experimental groups were composed of females and males between 50 and 89 days of age with an average weight of 20.9 g. All animals received food and water ad libitum.

The animals were housed in the animal facility of the Faculty of Medicine of the University of Alcalá, according to current regulations for the use of experimental animals. The animal model selected for this work was the C57BL/6J mouse due to its good maintenance and handling, as well as its pigmentation characteristics. 

Before the experiments, the circadian rhythm of the animals was maintained by exposure to white light and darkness in periods of 12 h/12 h, respectively, for a week. 

### 4.2. Cages for White and Violet Illumination

**Figure 4:**
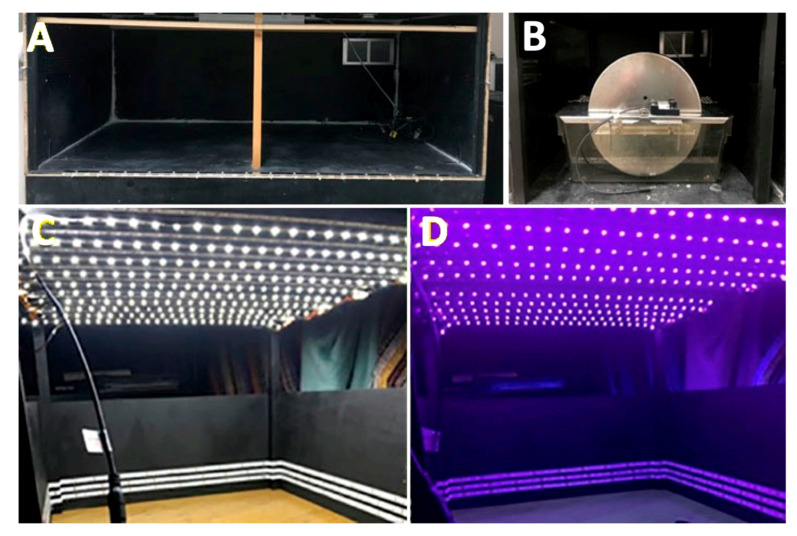
Cages for evaluating the circadian activity under different light conditions. (**A**) An example of an empty box. (**B**) An example of an activity wheel introduced into the box. (**C**) An example of the space inside the box illuminated with white light. (**D**) An example of the space inside the box illuminated with violet light.

To carry out the experiments under different wavelength exposures, it was necessary to adapt the spaces (Figure 4). Closed boxes (110 × 58 × 38 cm) without any windows and with a ventilation grill with a stable degree of humidity were built. Box walls were dark-painted to avoid reflections that could be caused by light sources. Four activity cages were placed inside each box and each cage was equipped with an activity wheel with rotation sensors. Each cage contained a single animal. Methacrylate cages measuring 16 × 36 cm and 14 cm high were used (Royal Decree 53/2013 on the protection of experimental animals).

The cages were placed in such a way that each of their sides and the top of the wheel were maintained 4.5 cm from the different wooden walls. The walls and ceiling of these boxes were arranged with LED strips of white light and violet lights to perform the different lighting requirements for each experiment. Each strip was 5 m long, integrating 300 pieces (LEDs).

In the case of white light, LED strips were fixed to the walls and the ceiling, achieving an illumination of 286 lux. In the case of violet light, LED strips were placed on the walls and ceiling achieving an illumination of 16 lux. The strips of white light and violet light LEDs were interspersed. 

**Figure 5:**
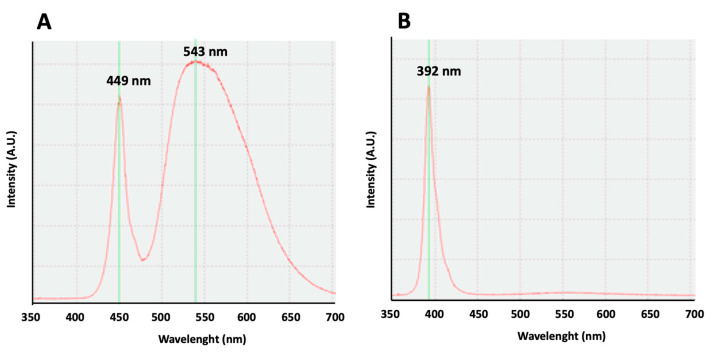
Light emission curves. (**A**) An emission curve of the white light source. Two maximum peaks may be observed at 449 and 543 nm. (**B**) An emission curve of the violet light source. A maximum peak can be observed at 392 nm.

For the emission of white light, LED sources were used (model ON-DT31-DW-EU-NF (ONFORU)), with a power of 12 W, a high color temperature (6000 K), and two maximum peaks at 449 and 543 nm (Figure 5A). For the emission of violet radiation, LED sources (model ELSAU004201 (ONFORU)), with a power of 12 W, were used. The emission curve of these violet radiations appears with a main peak at 392 nm (Figure 5B).

The light intensity inside the cage was recorded with a luxometer in cd/m^2^ and luxes in different locations, including the center of the cage. To characterize the emitting sources of violet radiation and white light, as well as the transmittance of the media (cage walls and filters), the Ocean Optics USB2000+ spectrophotometer was used. This spectrophotometer consists of a 2 MHz analog (A/D) converter, programmable electronics, a 2048-element CCD array detector, and a high-speed USB. This spectrophotometer provides a resolution of 0.35 nm (FWHM). 

Finally, since photometric quantities are not appropriate for describing the mice’s perception of lighting, the laboratory’s instruments cannot give values in radiometric units, thus photometric quantities are used in this paper and have to be considered with attention. 

### 4.3. Study Design

Different series of experiments were carried out. Animals were divided into five groups and maintained in different lighting conditions in the cages: 

Group 1. Four mice were exposed to white light and darkness in cycles of 12 h/12 h, respectively, for 30 days.

Group 2. Four mice were exposed to white light, violet radiation, and darkness in cycles of 12 h/4 h/8 h, respectively, for 30 days. 

Group 3. Four mice were exposed to violet light and darkness in cycles of 12 h/12 h, respectively, for 30 days.

Group 4. Four mice were exposed to white light and violet radiation in cycles of 8 h/16 h, respectively, for 30 days. 

Group 5. Four mice were exposed to white light plus violet radiation and violet radiation in cycles of 8 h/16 h, respectively, for 30 days. 

### 4.4. Measure of Animal Activity

The activity of the animals was measured using activity wheels (rotameter) useful to quantify the time that the mice were active. These wheels are made of stainless steel, and the rotameter has a diameter of 36 cm and 10 cm wide. In addition, the rotameters incorporate a magnetic sensor that captures the number of rounds and transmits the information to the magnetic revolution counter which, together with the Programmable Multicounter model LE3806, (Panlab-Harvard Apparatus, Cornella de Llobregat, Barcelona, Spain) obtains the data that demonstrate the circadian activity of the experimental animal. 

### 4.5. Electroretinography

The retinal functional response was evaluated by electroretinogram (ERG) recording. The ERG recordings were performed on dark-adapted animals after animal anesthesia induction under dim red light. The animals were anesthetized by intraperitoneal injection of a combination of ketamine (70 mg/kg) and xylazine (7 mg/kg), diluted in saline solution (NaCl, 0.9% in distilled water). 

Anesthetized animals were placed inside a Faraday cage to avoid external electromagnetic interference during recording. The animal’s temperature was maintained at 37 °C using a heating blanket (Hot-Cold, Pelton-Sherpherd Industries, Stockton, CA, USA). Their pupils were dilated with single drops of 1% Tropicamide (Alcon Cusí, SA, El Masnou, Barcelona, Spain). In addition, single drops of methylcellulose (2% Methocel, Omnivision, Neuhausen, Switzerland) were administered to protect the cornea and improve signal conductivity. 

For the ERG recordings, three electrodes were placed on the mouse. First, a 30G needle was inserted subcutaneously into the animal’s tail, which served as a ground electrode. The reference electrode consisted of a tab-shaped gold plate that facilitates placement in the oral cavity of the animal. Finally, the corneal or recording electrode, which was made of transparent methacrylate and gold was placed in the visual axis, locating it approximately 1 mm from the cornea (Burian-Allen commercial electrode, Hansen Ophthalmic Development Lab, Coralville, IA, USA). This corneal electrode, in the shape of a contact lens, was manipulated using holding support and was placed in contact with the Methocel gel previously placed on the cornea.

The signals were recorded from the right eye under scotopic conditions in response to uniform light stimulation applied by the Ganzfeld dome. Light flashes in the range of −4.0–2.0 Cd·s·m^−2^ were applied, and at least 10 signals per stimulus were averaged. The illumination provided by the Ganzfeld dome source was measured using a Gossen photometer (Nürnberg, Germany). 

The signals were amplified 1000 times by means of a Grass amplifier (CP511 AC amplifier, Grass Instruments, Quincy, MA, USA) and were filtered with a band-pass established between 0.3 and 1000 Hz. The amplitudes of the different components of the ERG waves were measured manually using the commercial software LabChart Pro v.8.1.13 (ADInstruments Ltd., Oxfordshire, UK). 

### 4.6. Immunohistology

Animals were sacrificed by intraperitoneal injection with approximately 25 mg. pentobarbital (Dolethal, Vetoquinol, Madrid, España) after the end of the light exposition protocols. After separating the eyelids and conjunctiva with the help of curved forceps and microscissors, the extraocular muscles and tissues around the eyeball were cut, and the eyeball was removed and exposed to 4% paraformaldehyde for 1 h. After this time, the cornea was opened by cutting the anterior part, so that the 4% paraformaldehyde could penetrate the cornea, and it was left in the tumbler for 1 h at room temperature. After the crystalline lens was removed, the eyes were left again in the tumbler with 4% paraformaldehyde for 30′ at room temperature. Subsequently, three 10-min washes in PBS were carried out to remove the paraformaldehyde. Then, half of the sample was preserved in phosphate-buffered saline (PBS) and cryoprotected by immersion in increasing concentrations of sucrose (20%, 30%, and 40%) in PBS, and in agitation. The last step, before freezing, is to embed the eyes in the Optimal Cutting Temperature compound (OCT, Agar Scientific, Ltd., Stansted, Essex, UK), after removing the rest of the sucrose. Then, they were placed in molds, which were left on the cold plate of the cryostat for freezing with OCT. Subsequently, cross-sections were made with the 15-micron X cryostat (Leica CM1950, Leica, Switzerland), which were placed on the slides. After the procedure, slides with the samples remained at −20 degrees for their conservation. 

Tissue slides were unfrozen and stained for αPKC + DAPI following the next protocol: after 3 washes of 10 min each by stirring and preincubation with PBS + Triton x-100 0.2%, the sample was incubated with primary antibodies + PBS + 0.2% Triton X-100 + 2% serum overnight in a humid chamber. Rabbit polyclonal antibody anti-αPKC was used (1:100 dilution, AB_477345, Sigma-Aldrich, Darmstadt, Germany). Then, the triple wash of 10 min was repeated, and the sample was incubated with secondary antibodies + PBS + Triton X-100 0.2% + Serum (2%) for one hour and 30 min in a wet chamber. A secondary Cy^TM^3 antibody (1:200 dilution, AB_2307443, VITRO, Jackson, Cambridge, UK) was used. DAPI is then applied for nuclear staining. Finally, several washes are carried out in PBS (triple-washed for 10 min in stirring, and the assembly and lacquering of the sample is carried out).

Images were taken with confocal microscopy (LeicaTCS-SP5, Leica, Switzerland). The confocal images of retinal sections were studied in three different fields of each animal, and the measurement of the number of cell photoreceptors per column was addressed for each animal. Cell numbers of the three areas from the four animals of each lighting condition were averaged. 

### 4.7. Statistics

Differences among variables under different lighting conditions were analyzed. When a variable was compared between more than two groups, a one-way ANOVA was used. When more than one variable was compared between groups, a two-way ANOVA was used. When a variable was compared between two groups, a Student’s *t*-test was used.

## 5. Conclusions

Violet light alters the circadian rhythm and visual function in mice. However, this radiation does not produce changes in the structure of the retinas of exposed animals after four weeks of light exposure.

## Data Availability

The data presented in this study are available on request from the corresponding author.
